# Applications of CRISPR-Cas Technologies to Proteomics

**DOI:** 10.3390/genes12111790

**Published:** 2021-11-12

**Authors:** Georgii Dolgalev, Ekaterina Poverennaya

**Affiliations:** Institute of Biomedical Chemistry, 119121 Moscow, Russia; k.poverennaya@gmail.com

**Keywords:** proteomics, CRISPR-Cas, genome editing, interactomics

## Abstract

CRISPR-Cas-based genome editing is a revolutionary approach that has provided an unprecedented investigational power for the life sciences. Rapid and efficient, CRISPR-Cas technologies facilitate the generation of complex biological models and at the same time provide the necessary methods required to study these models in depth. The field of proteomics has already significantly benefited from leveraging the power of CRISPR-Cas technologies, however, many potential applications of these technologies in the context of proteomics remain unexplored. In this review, we intend to provide an introduction to the CRISPR-Cas technologies and demonstrate how they can be applied to solving proteome-centric questions. To achieve this goal, we begin with the description of the modern suite of CRISPR-Cas-based tools, focusing on the more mature CRISPR-Cas9 system. In the second part of this review, we highlight both established and potential applications of the CRISPR-Cas technologies to proteomics.

## 1. Introduction

Proteins and interactions between them constitute the largest portion of phenotype [1]. Nevertheless, relatively few known proteins have been assigned with well-studied biological function, and some protein-coding genes still have no satisfactory annotation whatsoever [2]. This situation is further complicated by the existence of enormous number of proteoforms that arise due to non-synonymous single nucleotide polymorphisms and the processes of alternative splicing and post-translational modification [3]. Frequently, proteoforms derived from a single protein-coding gene have different biological roles [4]. In the recent years, methods of proteomics such as mass-spectrometry and associated computational analysis have become substantially more accurate and sensitive, allowing for an improved level of detection of proteins and proteoforms at large scale [5]. However, in order to productively use these methods to study protein function, relevant biological models such as knock-out or knock-in cell lines are required.

An ability to introduce changes directly to the genome of cells provides a robust way of generating such models. Before the advent of CRISPR-Cas technology, several classes of genome editing instruments such as zinc finger nucleases (ZFNs), transcription activator-like nucleases (TALENs) and meganucleases had been successfully used for this task [6]. However, each of these classes had considerable limitations that hindered their widespread adoption [7]. Additionally, these instruments rely on protein-DNA interactions to recognize DNA targets, which requires engineering of new protein domain(s) for each new target site. Despite numerous advances in the field of protein engineering and significant optimizations of these specific systems, genome editing remained technologically challenging until the emergence of CRISPR-Cas technology.

The most fundamental feature of CRISPR-Cas systems is their use of RNA for target recognition [8]. Accordingly, retargeting CRISPR-Cas to any new site is as easy as expressing a new guide RNA molecule. This not only significantly simplifies the experimental setup, but as a consequence allows for fast iteration over experimental designs for accelerated development of new, more efficient CRISPR-Cas systems. Since the initial applications of CRISPR-Cas to genome editing in 2013 [9,10], the suite of CRISPR-Cas-based instruments has grown remarkably to include diverse applications ranging from genome-wide knockout screening [11] to RNA editing [12] and genome architecture engineering [13].

The potential of CRISPR-Cas applications specifically to proteomics has been long recognized [14]. Since these observations, however, both genome editing and proteomics have developed significantly. A considerable number of studies that feature a combination of CRISPR-Cas with the methods of proteomics have been published, which permits the examination of common practices. However, many other established genome editing applications are yet to be combined with the techniques of proteomics. To facilitate novel applications of the CRISPR-Cas technologies to proteomics, we begin this review with a broad overview of the modern CRISPR-Cas-based instruments to demonstrate best practices and possible applications to examining protein function. In the next part, we highlight current applications of the CRISPR-Cas technologies to proteomics and later discuss the upcoming developments in this subfield.

## 2. Overview of CRISPR-Cas Technologies

In nature, CRISPR (clustered regularly interspaced short palindromic repeats) together with CRISPR-associated (Cas) proteins form a prokaryotic adaptive immunity system that can remember, recognize and neutralize foreign genetic elements such as those derived from invading viruses or plasmids [15]. All CRISPR-Cas systems share the same fundamental mechanism of action [8]. After the integration of small segments of foreign genetic elements in host DNA, these segments are transcribed as part of guide RNA molecules and ultimately govern the recognition and cleavage of invading genetic material.

At each step of this mechanism, different CRISPR-Cas systems display significant variability in terms of involved structures and mechanisms [16]. The most recent classification of CRISPR-Cas systems organizes them in 6 types and 33 subtypes, however, these numbers are expected to grow significantly in the coming years [17]. Due to the biotechnological potential of applied CRISPR-Cas systems, considerable resources are being devoted to analyzing genomic and metagenomic data in order to discover new CRISPR-Cas systems that may have advantageous traits [18].

CRISPR-Cas9 system was the first CRISPR-Cas system to be applied to genome editing in animal cells [9,10]. Since then, CRISPR-Cas technology has expanded to include various modifications of the CRISPR-Cas9 and alternative systems developed from other CRISPR types [19]. However, an original form of CRISPR-Cas9 system that features Cas9 nuclease from *Streptococcus pyogenes* remains by far the most popular CRISPR-Cas system for genome editing to date. It is extensively used for studying functions of proteins, for instance, to generate gene knockouts for loss-of-function studies [20] or to introduce point mutations in the coding frame of a gene to assess their functional significance [21]. Appropriately, we focus our discussion on the CRISPR-Cas9 and later introduce other systems as alternatives or additions.

### 2.1. Genome Editing with CRISPR-Cas9-Mediated Introduction of Double-Strand DNA Breaks

Cas9 nuclease from *Streptococcus pyogenes* (SpCas9) belongs to type II CRISPR-Cas system and is a single subunit protein with the molecular weight of 162 kDa [22]. The most significant structural feature of Cas9 is the presence of two nuclease domains, HNH and RuvC [23]. In the absence of guide RNA, Cas9 exists in an auto-inhibitory conformation, which prevents DNA binding and nuclease activity.

The RNA part of CRISPR-Cas9 system is represented by a single guide RNA (sgRNA), which contains a scaffold structure that facilitates binding by Cas9 and a spacer sequence that is complementary to a target DNA site. sgRNA is an artificial construct that has been engineered to mimic naturally occurring complex of CRISPR RNA (crRNA) and transactivating CRISPR RNA (tracrRNA), which is a characteristic feature of type II CRISPR systems [9]. While using sgRNA is the most popular strategy, several specific applications such as in vivo genome editing can benefit from using crRNA:tracrRNA complex instead [24].

Cas9 and corresponding sgRNA can be delivered to the target cell via a variety of methods. One of the most widespread approaches is plasmid-driven coexpression of Cas9 and sgRNA, which can be achieved via liposome-mediated transfection or viral transduction of the plasmid(s) [25]. Alternatively, purified Cas9 protein and sgRNA can be assembled in vitro and then delivered as a ribonucleoprotein complex (RNP) to the cell [26]. There are many additional methods of Cas9 and guide RNA delivery either in the form of plasmid or RNP that provide higher delivery efficiency for specific cells [27]. In some cases, such as when working with human primary cells or iPSC, the delivery of CRISPR-Cas components is a rate-limiting step for genome editing experiments.

Once active Cas9-sgRNA appears in the target cell, it scans the genome in search for a PAM site, which is NGG (N—any base) in case of SpCas9 [28]. Importantly, SpCas9 can also recognize NAG PAM, albeit with much lower efficiency [29]. PAM recognition by Cas9 is facilitated by DNA-protein interactions [28]. The recognition of PAM triggers a conformational change of the nuclease, which aligns the spacer sequence of sgRNA with PAM-adjacent DNA sequence on the complementary strand [22]. If sufficient level of complementarity between these sequences is achieved, the second conformational change exposes the target DNA to HNH and RuvC nuclease domains, which results in a double-strand DNA break (DSB) three bases upstream from PAM [23].

The resulting DSB can be repaired via two major pathways: non-homologous end joining (NHEJ) and homology-directed recombination (HDR) [30]. These two mechanisms are exploited for different strategies of genome editing with CRISPR-Cas9.

#### 2.1.1. Generating Gene Knockouts with NHEJ-Mediated Repair of DSBs

Loss-of-function experiments provide one of the most fundamental approaches to determining gene significance and function [20]. Before the advent of CRISPR-Cas technology, RNA interference was used to silence gene expression at the level of RNA (gene knockdown) [31]. However, RNA interference is prone to off-target effects and incomplete levels of gene silencing [32]. Genome editing allows to introduce mutations such as a frameshift or large deletions directly to the gene sequence, which renders the resulting protein product nonfunctional (gene knockout).

Efficient generation of gene knockouts can be achieved by leveraging NHEJ-mediated repair of DSBs. NHEJ is the dominant pathway for repairing DSBs in most animal cells [30]. Importantly, NHEJ is an error-prone process that frequently leads to small deletions or insertions (indels) at the site of the DSB. Even if a round of repair is error-free, Cas9 will be retargeted to the site, which will likely lead to an indel after a few rounds of repair. Additionally, large gene deletions can be generated by simultaneous targeting of distinct PAMs in the gene frame [33].

While the nature of resulting indels after NHEJ-mediated repair is inherently stochastic, possible outcomes can be predicted on the basis of the structure of the local DNA sequence [34].

#### 2.1.2. Generating Gene Insertions with HDR-Mediated Repair of DSBs

While obtaining gene knockouts with stochastic indel generation is a robust strategy for determining gene function, an ability to introduce arbitrary changes to the genome is far more powerful. The range of applications of this ability to functional studies is potentially limitless, but salient use cases include endogenous gene tagging [35] and introduction of functionally significant mutations into genes [21].

Introducing arbitrary mutations to the genome is possible with HDR-mediated repair of DSBs [9]. If a DNA sequence with sufficient homology to the target region is present within the cell during the repair, it can be utilized as a template for an HDR-mediated error-free repair of the DSB. Consequently, if the repair template carries an insertion or other mutation, the mutation will be integrated at the target site. Naturally, the mechanism of HDR can be used instead of NHEJ to introduce mutations which result in gene knockout [36], however, HDR-mediated approach can be less efficient (see below).

The nature of the repair template of choice primarily depends on the length of the desired mutation. Plasmid-based donors are straightforward, affordable and provide adequate insertion efficiency, especially in cases of insertions larger than 1 kb [25]. Notably, linearized plasmids or other large linear dsDNA molecules demonstrate higher editing efficiency compared to circular DNA donors [37]. For smaller insertions, single-strand oligodeoxynucleotide (ssODN) is the most efficient choice for insertions smaller than 200 bp [25], while larger ssDNA molecules are recommended for insertions of 200–1000 bp [38]. The choice of donor construct can also be influenced by other conditions of experiment, so it is important to select the most appropriate construct for each genome editing experiment [39].

In animal cells, the process of HDR-mediated repair is significantly less active than NHEJ-mediated repair [40]. Multiple strategies have been devised to increase the efficiency of HDR [41,42]. One approach has been based on the fact that HDR is only active in S and G2 phases. Synchronization of the cell cycle with reversible chemical inhibitors of the cell cycle phases and subsequent timed delivery of CRISPR-Cas9 with a donor sequence leads to significantly higher levels of cells with the desired insert [43]. Another group of approaches relies on the inhibition of NHEJ via delivery or expression of specific inhibitors of key components of the NHEJ pathway [44]. Next, Cas9 can be fused to some domains of DSB repair proteins to stimulate HDR [45]. The last, but not the least set of strategies employs donor sequences with altered structures, such as asymmetric ssODNs [46] or double-cut plasmid donors [47].

It is worth addressing here that HDR is not the only mechanism that can be exploited for generating insertions. The NHEJ machinery has been exploited for C-terminal epitope tagging (CRISPaint) [48], while microhomology-mediated end joining (MMEJ) mechanism is used to introduce small insertions in the genome [49]. While these approaches can provide increased editing rate in specific use cases, HDR-based approach is usually sufficient and remains more widely applicable.

By utilizing an appropriate strategy for a desired mutation, it is possible to achieve > 50% rate of successfully edited cells. A comprehensive and well-written guide to selecting the most effective strategy for CRISPR-Cas9-mediated insertion can be found elsewhere [39]. 

#### 2.1.3. DNA Targeting Range of Cas9

The targeting repertoire of Cas9 and other Cas nucleases is limited by the requirement of PAM. Simple calculations indicate that SpCas9 has, on average, one possible target site for every 8 base pairs of human genome (if only NGG PAM is taken into consideration). However, due to the inherently uneven distribution of PAM sequences across the genome, some regions of interest may lack an appropriate PAM, especially if high precision is required. For applications like HDR-mediated insert, the least possible distance between the DSB and the location of the desired insert is recommended for the best efficiency [50].

To increase the reach of genome editing with CRISPR-Cas, Cas nucleases with alternative PAM specificities are desired. Unsurprisingly, many Cas9 orthologues that recognize different PAMs have been studied and adapted to genome editing in human cells. For instance, Cas9 from Streptococcus thermophilus (StCas9) recognizes PAM of NNAGAAW (W stands for T or A) [9], while Cas9 from Streptococcus aureus (SaCas9) recognizes PAM of NNGRRT (R stands for A or G) [51]. Importantly, these Cas9 orthologues provide similar levels of genome editing activity in animal cells compared to SpCas9.

A different approach for obtaining nucleases with altered PAM specificities is based on engineering SpCas9 variants with mutated PAM-recognizing domain. With the help of sophisticated computational structural analyses and methods of directed evolution, several SpCas9 variants with a different PAM requirement have been developed [52]. 

While the necessity of PAM remains a fundamental limitation of the CRISPR-Cas systems, it is estimated that soon the target range of available Cas nucleases will almost completely cover the human genome [53].

#### 2.1.4. Specificity and Off-Target Effects of Cas9

One initial consideration raised from the first applications of Cas9 to genome editing was its pronounced off-target activity [54]. Worryingly, indels were detected even in the regions of DNA which had multiple mismatches with the spacer portion of the sgRNA. Such changes can affect expression levels and functioning of proteins, thus leading to possible incorrect conclusions about the biological problem in question. Accordingly, multiple studies have been conducted to find the principles underlying the off-target activity of Cas9 [55,56].

Most of these investigations employed libraries of sgRNAs that had mismatches to the analyzed target site. By correlating the efficiency of DNA cleavage in this site with selected features of sgRNAs, it was possible to calculate rules that can help select the sgRNA with the lowest off-target activity. More high-throughput methods for detecting genome-wide off-target effects of sgRNAs, such as GUIDE-seq [57] have provided even more data regarding the specificity of Cas9. Most of the popular software solutions for selecting sgRNA now incorporate the results of these studies and will rank candidate sgRNA accordingly [58].

For very sensitive applications, multiple engineered Cas9 variants with dramatically increased specificity such as eSpCas9 [59], HiFi Cas9 [60] and Sniper-Cas9 [61] are available. The genome editing efficiency and specificity of these and multiple other Cas9 variants have been recently compared [62].

#### 2.1.5. Efficiency of Cas9-Mediated DNA Cleavage

Another important concern when selecting sgRNA for an experiment is the resulting activity of sgRNA. As was discovered, different sgRNAs can perform very differently depending on the composition of the nucleotide sequence of sgRNA [63]. Mirroring the studies of off-target activity of sgRNAs, extensive libraries of sgRNA were tested to find correlations between their features and their activity [56,64]. While several empirical rules have been made based on these studies, manual prediction of sgRNA efficiency remains challenging and most of the resulting data was used to train predictive models that are now employed by common software for selecting sgRNA [58].

#### 2.1.6. Alternative Nucleases from Other CRISPR Types

Several nucleases from other types of CRISPR-Cas systems represent interesting alternatives to the CRISPR-Cas9.

Cas12a nuclease (also known as Cpf1) from the type V CRISPR system is a monomeric protein that acts similarly to Cas9 [65]. In contrast to Cas9, Cas12 possesses an ability to process crRNA arrays itself, which can simplify multiplexing experiments [66]. Additionally, DNA cleavage by Cas12a produces sticky ends, which can be useful for some experimental strategies. The most studied Cas12 nucleases belong to bacteria *Acidaminococcus* spp. (AsCas12) and *Lachnospiraceae* spp. (LbCas12) and recognize PAM of TTTV (where V is A, C or G) [65].

Other Cas nucleases with interesting properties are actively being discovered. For example, AsCas12f1 from *Acidibacillis sulfuroxidans* is the smallest known Cas nuclease (422 amino acids) that is active in animal cells [67]. As a comparison, the smallest discovered Cas9 orthologue to date, CjCas9 from *Campylobacter jejuni*, has a length of 984 amino acids [68].

### 2.2. Genome-Wide Screening

One of the most powerful applications of CRISPR-Cas9 system was the development of methods for high-throughput genome-wide screening studies [69]. Due to the advances in oligonucleotide and cDNA synthesis, libraries that contain 3 and more sgRNAs per every human gene were created [69,70,71]. With the help of these libraries, it became possible to conduct genome-wide loss-of-function studies. This approach has been used extensively to map genetic dependencies of cancer lines [72], for systematic discovery of genetic interactions [73] and for elucidation of resistance factors to particular therapies [74].

### 2.3. Other Notable CRISPR-Cas Technologies

In the recent years, a plethora of alternative CRISPR-Cas systems with novel functionality have emerged. Their development is made possible primarily by the realization that catalytically inactive Cas9 (dCas9) becomes a versatile RNA-guided DNA-targeting platform. This platform can be fused together with various effector domains, thus directing their activity to the DNA region of interest. Additionally, CRISPR-Cas systems that can act on RNA are also being actively introduced.

#### 2.3.1. CRISPR Interference

Double-strand DNA breaks are necessary for the action of CRISPR-Cas9. However, multiple DSBs can have adverse effects, such as activation of p53 and subsequent cell death [75]. CRISPR interference (CRISPRi) refers to a group of CRISPR-Cas technologies based on dCas9 that can silence the target gene without introducing DSBs.

The first system of this class relied on targeting dCas9 to a gene’s ORF, thus blocking the RNA polymerase from advancing and leading to inhibition of transcription [76]. Soon after the development of this system, much more efficient CRISPR inhibitors were introduced. A distinguished example is a complex of dCas9 fused to a transcription repressor factor Krüppel-associated box (KRAB) [77]. By targeting this complex to promoter or enhancer regions, efficient silencing of associated gene can be achieved.

One particular disadvantage of these CRISPRi systems is the need for constant expression of its components to maintain the gene silencing. This need can be alleviated by fusing DNA methyltransferase domains to dCas9-KRAB complex [78]. Such complex promotes heritable epigenetic silencing of a target region that remains for multiple cellular generations. The most recent system of this type, CRISPRoff, demonstrated an ability to silence gene expression almost completely (>90% reduction in expression) for the duration of at least 50 days [79].

#### 2.3.2. CRISPR Activation

CRISPR activation (CRISPRa) is based on the same principle as CRISPRi, but rather than recruiting transcriptional repressors to silence the target gene, these tools recruit transcriptional activators to drive the gene overexpression. The first system of this kind was based on fusing a VP64 domain (four repeats of the herpes simplex VP16 activation domain) to dCas9 [80].

Since this initial result, a plethora of more potent CRISPRa systems have been developed [81]. Among them, a few can be highlighted as the more mature and efficient approaches [82]. One logical improvement over baseline dCas9-VP64 system is an addition of multiple other transactivation domains, such as p65 (transcription activation domain of the nuclear factor-κB transactivating subunit) and Rta (Epstein-Barr virus R transactivator). This approach is exemplified by dCas9-VPR system [83]. Another method, dCas9-SunTag, is based on engineering a special “tail” for dCas9 that functions as a scaffold for recruiting multiple transactivators [84]. Importantly, SunTag system can be used not only for CRISPRa, but for any other application with effector domains of choice. Finally, one more approach to increasing the efficiency of transcriptional activation is the engineering of special secondary structure of non-spacer portion of guide RNA. The secondary structure serves as an aptamer that binds an MS2 protein which in turn recruits transcriptional activators. This system, dCas9-SAM (Synergistic Activation Mediator) is considered to be the most effective among other second generation CRISPRa approaches for overexpressing a single gene [85].

#### 2.3.3. Base Editing & Prime Editing

Both dCas9 and Cas9 nickase (Cas9n) have been used to create base editors, which change single nucleotides in the genome without introducing DSBs [86]. This is achieved by fusing dCas9 or Cas9n with either cytidine base editors that can perform C -> T (G -> A) conversions [87] or adenine base editors that catalyze A -> G (T -> C) conversions [88]. Base editors catalyze conversions in a short, typically 5-bp region inside the spacer sequence.

Base editing is considered to be a very important development for in vivo genome editing, since for these applications high specificity and low level of adverse influence such as an introduction of DSBs are required [89].

Base editing can be used as an alternative for a more traditional Cas9-based gene knockout strategy. CRISPR-STOP, a strategy for editing the ORF of the gene to include premature stop codons, is one of such approaches [90].

Moreover, base editing can be used to control the program of alternative splicing by excluding specific exons. This is achieved by editing intron-flanking sequences, as exemplified by CRISPR-SKIP [91].

Prime editing is mechanistically different from base editing and has also been developed for the purpose of introducing small mutations without DSBs and donor DNA [92]. It is based on Cas9 nickase fused with engineered reverse transcriptase. Specific prime editing guide RNA (pegRNA) molecule both specifies a target DNA site and carries the desired edit. Prime editing can efficiently introduce any point mutations and small insertions or deletions in the genome. Doubtlessly, prime editing is one of the most interesting CRISPR-Cas technologies that has great potential for clinical applications.

#### 2.3.4. RNA Targeting

Editing potential of CRISPR-Cas is not restricted to DNA. The first developed CRISPR-Cas system to target RNA, RCas9, is based on modified CRISPR-Cas9 [93]. Cas9 can naturally cleave RNA, but due to the requirement of PAM it requires a special oligonucleotide called PAMmer that hybridizes with a target segment of RNA and mimics double-stranded PAM structure. Additionally, catalytically inactive RCas9 was applied to live-imaging of specific transcripts [94].

Some naturally occurring CRISPR-Cas types target RNA rather than DNA. Several Cas13 nucleases from type VI CRISPR-Cas systems have been adapted to programmable RNA targeting in animal cells [95,96]. Remarkably, transcript knockdown with Cas13 has consistently shown higher knockdown levels than the most efficient shRNA available [96]. Based on catalytically inactive Cas13, systems for adenine to inosine (REPAIR) [12] and cytosine to uridine base editing of RNA (RESCUE) [97] were developed. Targeting of pre-mRNA with Cas13 was also utilized for programmable exon exclusion and inclusion with CasFX system [98].

### 2.4. Summary of CRISPR-Cas Technologies

**Figure 1:**
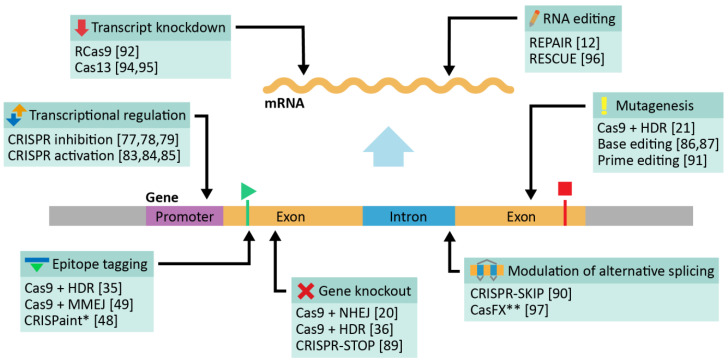
Applications of CRISPR-Cas. Note that illustrated locations and applications of CRISPR-Cas editing are not exhaustive. * CRISPaint only supports C-terminal tagging, but is otherwise similar to other methods of epitope tagging. ** CasFX acts at the level of pre-mRNA rather than DNA.

Providing a comprehensive survey of functionality of all available CRISPR-Cas technologies lies outside of the scope of the present work and information about additional CRISPR-Cas systems can be found in other excellent reviews [19,81,86]. All previously discussed systems, however, can be ultimately applied to studying protein function in combination with the methods of proteomics, which is the focus of this review. A schematic illustration of the most common applications of relevant CRISPR-Cas systems to genome and transcriptome editing is provided in Figure 1.

### 2.5. CRISPR-Cas in Proteomics

Genome editing technologies play an important part in the maturation of proteomics. Firstly, many advanced methods of proteomics now utilize CRISPR-Cas as an important part of their workflow. Additionally, biological mass spectrometry is being successfully applied to an increasing number of biological models of different levels of complexity, many of which have been developed with the help of genome editing technologies. Consequently, we find it appropriate to include both methods of proteomics and the generation of cellular models with subsequent proteomic analysis in the discussion of the applications of the CRISPR-Cas technologies to proteomics.

**Figure 2:**
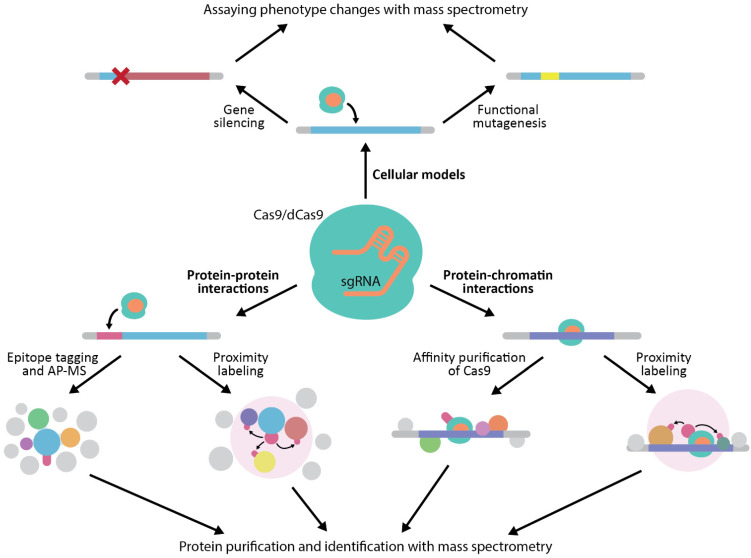
An overview of applications of CRISPR-Cas to proteomics experiments.

The current applications CRISPR-Cas to proteomics may be grouped in three major distinct groups (Figure 2): studying protein-protein interactions, studying protein-chromatin interactions and generation of cellular models. Applications to studying protein-protein and protein-chromatin interactions have been mostly created by adding CRISPR-Cas step to existing methods. However, addition of CRISPR-Cas allowed for specific advantages and more overall robustness of the methods. Generation of cellular models, on the other hand, consists of many novel methods for studying gene function in endogenous setting.

#### 2.5.1. Protein-Protein Interactions

Determining interaction partners of a particular protein is one of the most powerful approaches to analyzing the protein’s function [99]. A variety of different methods have been developed for this task [100].

One of such methods is affinity purification with mass spectrometry (AP-MS). Since most protein-protein interactions (PPI) are executed through protein complex formation, interaction partners of a particular protein can be specifically isolated from cells by affinity purification of the target protein itself. The identities of the interaction partners can be subsequently determined by mass spectrometry. Major advantages of AP-MS include its ability to study PPI in physiological conditions at large scale and decent levels of data reproducibility despite its complex workflow [101].

The choice of strategy for selective purification of a bait protein is a critical part of AP-MS experiments [102]. The most straightforward way to achieve selective purification of a protein from cells is to use antibodies. However, not all proteins have both potent and specific antibodies available [103]. One strategy to bypass this problem is to introduce predefined short peptide (tag) in the sequence of a target protein. This way, only one standardized antibody or affinity matrix is required for any number of proteins. The variation of AP-MS that includes tagging of the bait protein is known as tandem AP-MS (TAP-MS).

Traditionally, protein tagging has been accomplished with cloning protein cDNA in a vector that has tag sequence already included [104]. The resulting construct is then transfected or transduced into cells for the overexpression of the tagged protein. However, multiple studies indicate that overexpression can lead to protein mislocalization and high levels of non-endogenous binding [105,106]. Several strategies have been developed to provide a way for a more physiologically accurate expression level of tagged proteins. The best way to achieve an endogenous level of expression of a tagged protein is to insert nucleic sequence encoding the tag directly in the coding frame of the gene. This strategy is easily applied to lower eukaryotes such as yeast due to robust homology-directed recombination in their cells [107]. However, higher eukaryotes have significantly lower baseline activity of HDR, which makes knock-ins difficult to achieve and scale [108].

CRISPR-Cas provides an effective solution to this problem. For instance, Dalvai et al. developed a system for endogenous tagging of genes using CRISPR-Cas9 to use with AP-MS [109]. By using Cas9 nuclease to introduce DSB and a donor construct with homology arms of 300 bp, authors of the study managed to integrate 3xFLAG-2xStrep tag in the coding sequence of EPC1 and EP400 genes with the knock-in efficiency of 5–21%. The protein products of these genes were known to be a part of NuA4 complex. The following affinity purification and mass spectrometry experiments not only confirmed the stable association of EPC1 and EP400 in this complex, but also allowed to identify a novel subunit of NuA4. After proving the robustness of their method, authors attempted to address one major obstacle on the path to high-throughput application of the method, which is clonal selection. Selection and establishment of cell lines with the desired insert can take over a few weeks, which obviously limits the scalability of CRISPR-Cas-based knock-in methods. Authors hypothesized that with the observed knock-in efficiencies, sufficient levels of correctly tagged protein can be extracted from the cells without the process of clonal selection. Remarkably, the interactomic data obtained this way for EP400 protein showed high level of consistency with the previous data.

Stein et al. compared interactomic profiles of AMPKa2 subunit of AMP-activated protein kinase (AMP) obtained with Stable Isotope Labeling in Cell culture (SILAC) and Multidimensional Protein Identification Technology (MudPIT) in combination with either CRISPR-based tag insertion and subsequent affinity purification or direct immunoprecipitation [110]. While both approaches led to identification of some known interaction partners of AMPKa2, interactomic profiles obtained with TAP or IP showed little overlap. Notably, interactomic profiles obtained after IP with two different antibodies also demonstrated little similarity. The authors of the study concluded that CRISPR-Cas in combination with TAP can provide complimentary interactomic data to IP-based approaches, however, the accuracy of the latter heavily depends on the quality of available antibodies.

Recently, the first attempt to conduct a large-scale examination of the interactome of endogenously tagged proteins was published as a preprint [111]. OpenCell dataset includes interactomic profiles for 1261 genes, encompassing 30,293 interactions between 5271 proteins. Such large-scale examination was made possible due to the previous effort of this group to create tagging strategy with higher throughput than conventional approaches [112]. Most importantly, selected genes were tagged with a fluorescent protein. The correct fusions were identified on the basis of detectable fluorescence, thus bypassing the cumbersome process of clonal genotyping and expansion. One discovered limitation of this strategy was the need for relatively high expression levels of the fusion protein to achieve the necessary levels of fluorescence for sorting. As an additional improvement, authors used highly sensitive timsTOF mass spectrometers to dramatically reduce the amount of required input material. By comparing the obtained data against the curated database of protein interactions CORUM, it was concluded that OpenCell methodology produces more complete and accurate results than “non-endogenous” competing datasets.

While TAP-MS is a powerful strategy to discover protein interactions, it lacks an ability to capture transient or weak interactions, for example the ones that often happen between enzyme and substrate [113]. Since this kind of interactions can be very important to understanding protein function, another, complimentary method is required to detect them. Proximity labeling is a group of methods that has been created to fulfill this task [114]. The core of all proximity labeling methods is an enzyme that is fused to a bait protein and possesses a catalytic activity that can lead to covalent linkage of a small molecule to all proteins in the enzyme’s proximity. This small molecule can serve as a tag for affinity purification, and all purified proteins can be analyzed and identified by mass spectrometry.

A number of proximity labeling methods exist, each differing in the nature and catalytic mechanism of the labeling enzyme [115]. The first system of this kind, BioID, was released in 2012 and is based on the promiscuous biotin ligase BirA* [116]. This enzyme uses ectopically supplied biotin and cellular ATP to produce biotin-AMP, a highly reactive intermediate with short life-time. It is estimated that effective radius of proximity labeling with BirA* is 10 nm, with the maximum possible range of 30 nm [117]. Furthermore, biotin-AMP does not permeate membranes and thus maintains the compartmentalized nature of protein interactions. A major disadvantage of BioID is long effective labeling time of around 18 h. To address this, TurboID and miniTurboID systems were developed [118]. These enzymes are biotin ligases with effective labeling time of 10 min, which provides much better resolution than BioID. Aside from biotin ligases, another popular system is based on engineered ascorbate peroxidase (APEX) and related APEX2, which use H2O2 and biotin-phenol to produce biotin-phenol radicals that label adjacent proteins [119]. APEX enzymes have very short labeling time (1 min), but they are restricted for some applications due to the toxicity of H_2_O_2_.

BioID together with CRISPR-Cas9 were extensively used to study the interactome of important proteins of *Toxoplasma gondii*. In the first study of this kind, several proteins essential to growth and invasion were first identified with CRISPR-Cas9 knockout and auxin-induced degradation [120]. To understand the function of these genes, authors of the study used CRISPR-Cas9-mediated HDR-based technology to insert BirA* enzyme in the genome to create endogenous fusions of this enzyme with the studied genes. After the analysis of interaction partners of these proteins with mass spectrometry, it was concluded that another protein is necessary for their association and action. In another study, the same group of authors expanded upon their approach and analyzed the interactome of the newly identified markers to establish the connection map of parasitophorus vacuole, which may provide necessary information to develop effective counteracting drugs [121]. In the following year, the group published an updated protocol to apply BioID with CRISPR-Cas to *Toxoplasma gondii* [122].

Another interesting approach to apply BioID to studying the interactome of endogenous proteins was demonstrated by Vandemoortele et al. [123]. Using adenoviral delivery of targeting vector and standard HDR protocol, authors integrated BirA* plus T2A self-cleaving peptide at the N-terminus of p53. Due to the self-cleavage of T2A, the resulting protein product of this fusion gene is separated into intact p53 and BirA*. However, by targeting a BE3 base editor to the T2A sequence in the genome, it can be mutated and inactivated, thus leading to the appearance of p53-BirA* fusion in target cells. Unedited cells can then be readily used as a negative control. From this point, using standard BioID protocol, interacting partners of p53 can be discovered. By comparing the data obtained this way to the data gathered from experiments with ectopic expression of p53-BirA* fusion, authors concluded that their approach showed much higher accordance to the curated data and predicted functions of p53.

While the approach of studying the interactome of endogenously tagged genes is considered to be more accurate than alternatives [111,123], it is important to note that as of yet there has been no systematic investigation of the effect of the tagging strategies on the resulting quality of the interactomic data.

#### 2.5.2. Chromatin-Protein Interactions

Many proteins carry out their functions through interactions with DNA [124]. While the methods for isolation and analysis of genomic regions associated with a particular protein have been well developed even before the arrival of CRISPR-Cas [125], the reverse task of identifying all proteins bound to a specific locus is still considered to be much more challenging [126].

One group of approaches for analyzing locus-specific proteomes relies on the DNA targeting nature of dCas9. Addition of an affinity tag to dCas9 and subsequent affinity purification of dCas9-chromatin complexes from cells allows for extraction and analysis of chromatin-associated proteins. In 2013, Fujita et al. reported the development of engineered DNA-binding molecule-mediated chromatin immunoprecipitation (enChIP), a method that uses dCas9 fused with 3xFLAG tag to isolate specific locus [127]. After targeting this fusion to the specific DNA sequence, the associated region of the chromatin was extracted together with all bound proteins. The identities of the associated proteins were then determined with mass spectrometry. The same group later adapted enChIP for use in prokaryotes [128]. A similar method, CRISPR-based chromatin affinity purification with mass spectrometry (CRISPR-ChAP-MS), utilizes Protein A as an affinity handle [129]. Another technique of this kind, CRISPR affinity purification in situ of regulatory elements (CAPTURE), uses biotinylated dCas9 [130]. In a different approach termed Cas9 locus-associated proteome (CLASP), dCas9-3xFLAG is added in vitro to chromatin mixture generated from mechanical shearing of crosslinked cells, but the next steps of affinity purification and mass spectrometry are akin to the aforementioned methods [131].

As with protein-protein interactions, more sensitive detection of the proteins associated with a particular locus can be accomplished with proximity labeling [132]. The first system of this kind, CasID, featured the fusion of dCas9 with BirA* enzyme [133]. This system was tested on well-studied genomic regions such as telomeres and major satellite repeats, with the results being in agreement with previously published studies. APEX2 enzyme is another popular choice for chromatin proximity labeling. For instance, dCas9-APEX2 Biotinylation at genomic Elements by Restricted Spatial Tagging (C-BERST) is a method that was used to study the protein composition of human telomeres and centromeres [134]. The resulting data was found to be consistent with previous reports on protein composition of these regions. As indicated by the authors of the study, methods utilizing APEX2 offer a unique advantage of being able to capture rapid changes of chromatin structure due to the short labeling time of the enzyme. A few other groups have also developed alternative systems that utilize dCas9 and APEX2 for chromatin profiling [135,136].

The field of functional proteomics that studies nucleic acid-bound proteins is rapidly developing and new CRISPR-Cas-based systems for studying protein-chromatin interactions are being engineered, as indicated by a recent comprehensive review of this topic [137].

#### 2.5.3. Subcellular Proteomics

Determining protein localization is a vital step to understanding biological role of the protein [138]. Fluorescence microscopy is one of the most popular methods for protein localization studies [139]. Technically, fluorescence microscopy is not considered to be one of the methods of proteomics, but localization studies with fluorescence are often conducted at very large scale and it is agreed that such studies qualify for the definition of proteomics [140]. 

Fluorescent tags such as GFP permit live-cell imaging of the tagged proteins and are often used for large-scale localization studies [141,142]. CRISPR-Cas-mediated insertion of the fluorescent tag directly in the frame of the protein-coding gene can provide physiologically accurate protein localization data. The already discussed OpenCell dataset features localization data for 1311 human proteins, which was made possible by automatization of microscopy acquisition and subsequent rigorous manual assessment of protein localization [111].

#### 2.5.4. Generation of Cellular Models for Downstream Proteomics Analysis

The power of CRISPR-Cas genome editing is widely harnessed to create cellular models for assaying a phenotypic effect of a particular gene or a mutation. In these areas of research, methods of proteomics serve as complimentary methods of analysis together with other high-throughput methods of assaying phenotype such as RNA sequencing. 

Loss-of-function studies are de facto standard in the process of preliminary assessment of gene function [143]. By generating a cell line with a knockout of a target gene, its consequences can be thoroughly analyzed and gene’s significance and connection to the phenotype can be elucidated. Proteomics has been indispensable for these kinds of studies and together with RNA interference has already provided important functional characterization of many protein-coding genes [31]. However, CRISPR-Cas has recently replaced RNAi in such studies. For instance, Mehrabian et al. used Cas9 to generate knockout of the prion protein (PrP) in NMuMG epithelial cells and analyzed the phenotypic effect of the gene knockout with mass spectrometry [144]. The results of differential proteomics revealed the connection of PrP to cellular adhesion and differentiation, which indicated previously unreported potential functions of PrP. In the same vein, CRISPR-Cas9-mediated knockout coupled with quantitative proteomics has been used for studying functions of cancer-related genes such as GSTO1 [145] and FMRD6 [146].

Additionally, quantitative proteomics can be applied at both the exploratory phase of the study to identify relevant proteins and then at targeted level to study the phenotypic effect of CRISPR-Cas-mediated knockout of identified proteins. For example, this approach was used to identify and characterize biomarkers for predicting response of melanoma cells to immunotherapy [147].

CRISPR inhibition has been used in combination with quantitative mass spectrometry to study metabolic processes of *E. coli*. Landberg et al. used CRISPRi to silence several major genes that participate in purine and pyrimidine biosynthesis in *E. coli* and identified a few genes, which silencing led to growth decoupling and increased levels of production of single-domain antibody (sdAb) [148]. Proteomics analysis of the strains that have these genes downregulated showed that these strains maintain increased production levels by stopping cell growth and not reaching stationary phase as a result. Another group used similar approach of CRISPRi screening combined with quantitative mass spectrometry to study mechanisms that buffer downregulation of several important enzymes in *E. coli* [149].

Apart from loss-of-function studies, important disease-relevant models can be generated with precise mutations that can lead to a gain-of-function effect. These mutations induce significant phenotypic changes and can be readily studied with the methods of proteomics. For instance, differential proteomics and transcriptomics have been used to study the effects of EGFR C797S mutation, which is common in non-small-cell lung cancer (NSCLC) [150]. The mutation was introduced by CRISPR-Cas genome editing with HDR. The resulting data led to the discovery of genetic dependency of cell lines with EGFR C797S mutation, therefore indicating a possible mechanism of therapy.

CRISPR-Cas base editors are also being applied in combination with quantitative mass spectrometry. Chang et al. compared HDR-based approach and base editing for the correction of G2019S mutation in leucine-rich repeat kinase 2 (LRRK2), which is one of the most widespread genetic causes of Parkinsonism [151]. Adenosine base editing using the ABEmax system [152] demonstrated higher percentage of correctly edited clones (24.5% vs. 6.4%) and lower rate of off-target mutations (26.4% vs. 57.4%) than HDR-based strategy. The phenotypes of IPS cells with corrected mutation and control cells were compared with RNA-sequencing and mass spectrometry. Interestingly, although RNA-sequencing did not identify differentially expressed genes, multiple pathways were differentially regulated at the level of the proteome and the identified differences were consistent with both the observed phenotype and data from previous studies.

## 3. Prospects and Limitations

In this section, we will address present and future developments of CRISPR-Cas technologies with respect to the field of proteomics. Additionally, we will discuss some important considerations for applying CRISPR-Cas to proteomics.

### 3.1. Advances in Genome Editing

The most probable prediction about the future development of CRISPR-Cas based instruments is the increase in both specificity and efficiency [19]. While CRISPR-Cas systems already offer mostly streamlined and simple workflow, the ease of use of CRISPR-Cas systems will only be growing with time. For particular genome editing systems, large-scale studies of the efficiency of guide RNA and application-specific optimizations will lead to confident genome editing outcome for any target at least in the human genome [58]. For instance, while the efficiency of HDR-mediated insertion started in the range of 5–20% in the beginning of the CRISPR-Cas era, nowadays, with appropriate strategy, knock-in efficiencies of 60% and higher can be confidently achieved [39]. This leads to the rapid generation of both individual and large-scale models for subsequent analysis with the methods of functional genomics.

Moreover, CRISPR-Cas systems other than Cas9 will provide effective alternatives to some applications of the Cas9 and also offer completely new possibilities. This includes more potent CRISPRi [79] and CRISPRa [153] systems, more precise and efficient base editors [154] and many other applications such as RNA editing [155], locus visualization [156] and engineering chromatin architecture [13]. New CRISPR-Cas systems with advanced functionality are also regularly presented. For example, as we already mentioned, recently developed prime editing system offers the possibility of robust introduction of any point mutations or small insertions in the genome without introducing DSBs or requiring donor DNA [92].

### 3.2. Opportunities for Generating New Cellular Models for Proteomic Analyses

Even without all the recent advances in genome editing, the potential of genome editing coupled with proteomics is not utilized fully. Apart from straightforward loss-of-function studies, many disease relevant models were generated [157] and are yet not completely explored. We believe that the recent advances in biological mass spectrometry [158] and maybe even anticipated direct protein sequencing technologies [159] will lead to a wider adoption of methods of proteomics for characterizing these important cellular models.

Furthermore, for several advanced cellular models generated with CRISPR-Cas, these already is a groundwork for the use of the methods of proteomics to investigate these models. For instance, cellular reprogramming into pluripotency was recently achieved with CRISPR activation [160]. Previously, similar kind of transition was characterized by quantitative mass spectrometry [161]. From these examples, it is evident that many available complex cellular models can be readily studied with the methods of proteomics.

### 3.3. High-Throughput Genome-Wide Studies

Advances in both genome editing and proteomics will eventually lead to genome-wide characterization of protein functions at the endogenous level. At large scale, it will be largely achieved by genome-wide loss-of-function studies and genome-wide interactome profiling with AP-MS and/or proximity labeling. While the former already has established genome-wide libraries for efficient knockouts [70], the latter still awaits more robust methodology of library construction, with the most complete dataset up to date, OpenCell, featuring less than 15% of protein-coding genes [111]. Interestingly, one group recently developed a system for combined application of AP-MS and proximity labeling simultaneously [162]. While this system has not yet been applied in combination with CRISPR-Cas, this combination is likely a matter of time. Additionally, genome-wide studies will require mass spectrometry technologies with much higher throughput than what is available now. Recent advances in instrument sensitivity and multiplexing offer hope for soon solution to this issue [163]. One yet not discussed consideration for studying the endogenous functions of proteins is the simple fact that some proteins are poorly or even not expressed in available model cell lines. Potentially, this problem may be alleviated by CRISPR activation methods, but the influence of CRISPRa on the interactomic profile of upregulated genes has not yet been investigated.

### 3.4. Proteoforms

Perhaps the biggest yet insufficiently discussed consideration for applying methods of proteomics and CRISPR-Cas to study the endogenous functions of proteins is alternative splicing. Recently, it was established that alternative spliceforms can give rise to protein products with significantly different functionality [164]. Since many of the genome editing approaches to study protein function rely on the insertion of a tag sequence from either end of the open reading frame, it can theoretically lead to situations where alternative spliceforms with different biological roles are analyzed as one “protein”. Approaches relying on ectopic overexpression of proteins do not suffer from this problem due to the fact that the majority of these strategies rely on cloning cDNA, which means that usually only particular spliceform is selected for experiments.

Possible solutions to this problem include CRISPR-Cas-based modulators of alternative splicing either at the genome level [91] or at the level of pre-mRNA [98]. Hopefully, in the recent future, our understanding of transcriptional programs and advancements in gene editing will lead to the advent of versatile modulators of alternative splicing.

## 4. Conclusions

The discovery of the CRISPR-Cas systems has undoubtedly changed molecular biology, opening up new possibilities for studying biological processes and their components. At present, there is practically no area of life sciences left where the CRISPR-Cas technology has not been used. One of the most topical areas in modern biology is the width and depth of human proteome [165]. Many proteins that can have important or even vital functions are not yet comprehensively studied. Accordingly, studying human proteins at large scale will lead to quick progress in our understanding of human biology and disease. As demonstrated by the studies presented in this review, the potential of CRISPR-Cas technology to interrogate the functions of proteins in combination with the methods of proteomics provides robust framework for investigating protein function in detail. We hope that this review promotes larger adoption of CRISPR-Cas instruments for proteomics studies, which will ultimately lead to new and exciting discoveries.

## Data Availability

Not applicable.
